# Effectiveness of initiating extrafine-particle versus fine-particle inhaled corticosteroids as asthma therapy in the Netherlands

**DOI:** 10.1186/s12890-016-0234-0

**Published:** 2016-05-17

**Authors:** Thys van der Molen, Dirkje S. Postma, Richard J. Martin, Ron M. C. Herings, Jetty A. Overbeek, Victoria Thomas, Cristiana Miglio, Richard Dekhuijzen, Nicolas Roche, Theresa Guilbert, Elliot Israel, Wim van Aalderen, Elizabeth V. Hillyer, Simon van Rysewyk, David B. Price

**Affiliations:** Department of General Practice, University of Groningen, University Medical Center, Groningen, The Netherlands; Department of Pulmonary Medicine, University of Groningen, University Medical Center, Groningen, The Netherlands; National Jewish Medical and Research Centre, Denver, USA; Pharmo Institute for Drugs Outcome Research, Utrecht, The Netherlands; Research in Real Life, Ltd, Cambridge, UK; Radboud University Medical Centre, Nijmegen, The Netherlands; Groupe Hospitalier Cochin, AP-HP and University Paris Descartes, Paris, France; Cincinnati Children’s Hospital and Medical Center, Cincinnati, USA; Brigham and Women’s Hospital and Harvard Medical School, Boston, MA USA; Emma’s Children Hospital, Academic Medical Centre, University of Amsterdam, Amsterdam, The Netherlands; Observational & Pragmatic Research Institute Pte, Ltd, Singapore, Singapore; Academic Primary Care, University of Aberdeen, Polwarth Building, Foresterhill, AB25 2ZD Aberdeen UK

**Keywords:** Asthma, Effectiveness, Extrafine-particle, Fine-particle, Inhaled corticosteroids

## Abstract

**Background:**

Most randomised clinical trials typically exclude a significant proportion of asthma patients, including those at higher risk of adverse events, with comorbidities, obesity, poor inhaler technique and adherence, or smokers. However, these patients might differentially benefit from extrafine-particle inhaled corticosteroids (ICS). This matched cohort, database study, compared the effectiveness of extrafine-particle with fine-particle ICS in a real-life population initiating ICS therapy in the Netherlands.

**Methods:**

Data were from the Pharmo Database Network, comprising pharmacy and hospital discharge records, representative of 20 % of the Dutch population. The study population included patients aged 12 − 60, with a General Practice-recorded diagnosis for asthma (International Classification of Primary Care code R96), when available, ≥2 prescriptions for asthma therapy at any time in their recorded history, and receiving first prescription of ICS therapy as either extrafine-particle (ciclesonide or hydrofluoroalkane beclomethasone dipropionate [BDP]) or fine-particle ICS (fluticasone propionate or non-extrafine-particle-BDP). Patients were matched (1:1) on relevant demographic and clinical characteristics over 1-year baseline. Primary outcomes were severe exacerbation rates, risk domain asthma control and overall asthma control during the year following first ICS prescription. Secondary outcomes, treatment stability and being prescribed higher versus lower category of short-acting β2 agonists (SABA) dose, were compared over a 1-year outcome period using conditional logistic regression models.

**Results:**

Following matching, 1399 patients were selected in each treatment cohort (median age: 43 years; males: 34 %). Median (interquartile range) initial ICS doses (fluticasone-equivalents in μg) were 160 (160 − 320) for extrafine-particle versus 500 (250 − 500) for fine-particle ICS (*p* < 0.001). Following adjustment for residual confounders, matched patients prescribed extrafine-particle ICS had significantly lower rates of exacerbations (adjusted rate ratio [95 % CI], 0.59 [0.47–0.73]), and significantly higher odds of achieving asthma control and treatment stability in the year following initiation than those prescribed fine-particle ICS, and this occurred at lower prescribed doses. Patients prescribed extrafine-particle ICS had lower odds of being prescribed higher doses of SABA (0.50 [0.44–0.57]).

**Conclusion:**

In this historical, matched study, extrafine-particle ICS was associated with better odds of asthma control than fine-particle ICS in patients prescribed their first ICS therapy in the Netherlands. Of importance, this was reached at significantly lower prescribed dose.

**Electronic supplementary material:**

The online version of this article (doi:10.1186/s12890-016-0234-0) contains supplementary material, which is available to authorized users.

## Background

Traditional randomized controlled trials (RCT) aim to establish a clear cause-and-effect relationship between an intervention and an outcome. Although asthma RCTs have high internal validity, a problem is that they typically represent fewer than 5 % of patients with a current diagnosis of asthma [1, 2]. Patients at high risk of adverse events, with comorbid conditions such as obesity, rhinitis or smoking, or those with poor inhaler technique and adherence, are typically excluded. Real-life research can help balance the limitations of a RCT design [1]. Using real-life medical information recorded in primary care databases allows assessment of long-term outcomes in broader asthma populations cared for under usual conditions. These populations also include patients with unstable asthma often excluded from RCTs, but routinely seen by clinicians in primary care settings [3].

Traditional inhaler devices for controlling asthma symptoms produce fine particles with a mass median aerodynamic diameter (MMAD) of 2–4 μm; however, some newer pressurized metered dose inhalers (pMDI) using hydrofluoroalkane propellant generate an aerosol of smaller, extrafine-particles with an MMAD <2 μm [4, 5]. RCTs 8–12 weeks in duration have reported that extrafine-particle ICS is equally effective as fine-particle ICS in controlling asthma symptoms [6, 7]. Some patients typically excluded in these studies (see above) might differentially benefit from extrafine-particle ICS [4]. For these reasons, real-life database comparative effectiveness studies may usefully complement traditional RCTs to evaluate whether different properties of extrafine-particle and fine-particle ICS result in different effects in broader clinical populations.

Relevant real-life database studies in the UK and US show matched asthma patients prescribed extrafine-particle hydrofluoroalkane beclomethasone dipropionate (EF-HFA-BDP) for the first time had equivalent or better odds of achieving asthma control over 1 year than patients initiating fluticasone, despite being prescribed lower doses [8–10]. In contrast to findings from some RCTs [6, 7], these studies suggest that extrafine-particle ICS offers further clinical benefits in asthma control compared with fine-particle ICS therapy in real-life populations. To determine whether this finding can be generalized in other settings with different healthcare systems and possibly different prescribing habits, this real-life database study compared the effectiveness of initiating ICS therapy in patients with asthma in the Netherlands prescribed extrafine-particle ciclesonide or EF-HFA-BDP (Qvar®) versus fine-particle ICS fluticasone propionate or non-extrafine-particle-beclomethasone dipropionate (Non-EF-BDP). In line with previous real-life research [8–10], it was hypothesized that EF-ICS may be at least as effective as fine-particle ICS therapy.

## Methods

### Study design and data source

This was a matched cohort, database study, consisting of a baseline and outcome period. The baseline period served for patient characterization and confounder definition and was 1 year before the initiation date of ICS therapy. The outcome period was 1 year following the initiation date for evaluating the effectiveness of ICS therapy. The initiation date was the date when patients received their first prescription of extrafine-particle ICS (either ciclesonide or EF-HFA-BDP), or fine-particle ICS (either fluticasone or Non-EF-BDP). The study period was from January 1998 to December 2012.

An independent steering committee was involved in a priori development of study design, review of analyses and interpretation of results [11]. The study was registered with the European Network of Centres for Pharmacoepidemiology and Pharmacovigilance (ENCePP, study no. 8391), and conducted in accordance with the ENCePP Code of Conduct. The study design is summarized in Additional file 1: Figure S1.

Anonymous data to inform the study objectives were obtained from the Pharmo Database Network provided by the Pharmo Institute for Drugs Outcome Research (Utrecht, the Netherlands). The Pharmo Database Network comprises, among other databases, linked Outpatient Pharmacy, General Practice (GP), Hospitalization and Clinical Laboratory Register databases, and accounts for almost 3 million patients’, representative of 20 % of the Dutch population. The Outpatient Pharmacy database includes the dispensing records of more than 200 community pharmacies and is linked to hospital discharge records. Data from the Pharmo Institute were provided in accordance with Dutch privacy laws.

### Study population

The study population included adult patients aged 12–60 years with evidence of asthma, defined as ≥2 prescriptions for asthma therapy at any time in their recorded history, a General Practice-recorded diagnosis for asthma (International Classification of Primary Care [ICPC] code R96), when available, and receiving continuous ICS therapy following the initiation date, defined as ≥2 ICS prescriptions during the outcome period in addition to the first prescription. Patients were excluded from the study if, at any time, they had been diagnosed with any chronic respiratory diseases other than asthma. Patients prescribed long-acting muscarinic antagonists at baseline were not excluded.

### Outcomes

The rate of *severe asthma exacerbations in the year following ICS therapy initiation*, one of three co-primary outcomes, was defined based on the American Thoracic Society/European Respiratory Society (ATS/ERS) Task Force definition to include asthma-related hospital admissions or prescription for acute courses of oral corticosteroids. Asthma-related admissions were defined as any hospital entry for asthma or any lower respiratory reason (including lower respiratory tract infections). Acute oral corticosteroid use associated with asthma exacerbation treatment was defined as all courses that were not maintenance therapy, and/or all courses where dosing instructions suggested exacerbation treatment. Emergency department data were not included in the exacerbation definition as these data were not available in the database. Two further co-primary outcomes included *risk-domain asthma control*, defined as the absence of asthma-related hospital admissions and prescription for acute courses of oral corticosteroids; and *overall asthma control*, defined as achieved risk-domain asthma control and average daily dose of ≤200 μg salbutamol or ≤500 μg terbutaline.

Secondary outcomes were (1) *treatment stability during the outcome period,* defined as achieving risk-domain asthma control and no change in therapy; and (2) *average daily dose of short-acting β2 agonists (SABA)*, defined as $\frac{number\kern0.5em  of\kern0.5em  inhaler s\kern0.5em \times \kern0.5em  Doses\kern0.5em  per\kern0.5em  inhaler}{365}\kern0.5em \times \kern0.5em  strength,$ where *strength* is inhaler dose (μg). Change in therapy was defined as an ICS prescribed dose increase of ≥50 % or addition of new asthma therapy, including leukotriene receptor antagonists (LTRA), theophylline, or long-acting β-agonists (LABA).

Finally, two exploratory outcomes were (1) *prevalence of oral candidiasis*, based on the number and percentage of patients who either received a diagnosis of oral candidiasis in their hospital records, or the number and percentage of topical oral prescriptions for antifungals; and (2) *hospitalization rate*, defined as the number of any recorded hospital entry for asthma and/or any (asthma-related) lower respiratory reason in the year following the initiation date. All outcomes have been used in prior studies by the research group and have been described previously [8–10].

### Statistical analysis

The database extraction and statistical analysis plan were written before any analyses were conducted [12]. All analysis was carried out using IBM SPSS Statistics version 22, Microsoft Office EXCEL 2007, and SAS version 9.3. Complete details on statistical analysis methods are in the Additional file 1: Figure S1.

Patients were exact-matched (1:1) based on key baseline demographic and clinical characteristics to ensure comparison of similar patients and to reduce potential confounders [13]. Matching criteria were informed from baseline differences between treatment cohorts as evidenced by exploratory analysis (*t*-test/chi square test, *p* < 0.05), from expert clinical advice, and from previous research experience, and included the following: sex, age, exact year of ICS therapy initiation, severe exacerbations, LABA and LTRA prescriptions, and SABA daily dose. Patients were matched sequentially on each of the selected matching criteria. Patients who did not match were excluded.

The rate of severe exacerbations in the outcome period was compared between matched treatment cohorts using a conditional Poisson regression model. The odds of achieving risk-domain asthma control and overall asthma control, and changing asthma therapy, were compared between matched treatment cohorts using a conditional binary logistic regression model. Ordinal conditional logistic regression modeling was used to analyze prescriptions of higher doses of SABA (categorized doses). The models used empirical standard errors (for more conservative confidence interval [CI] estimations) and adjustments were made for any residual non-collinear baseline confounders (Pearson and Spearman correlation coefficients, *r* > 0.3) and for those variables predictive of the results through full multivariable analysis (multivariable model, *p* ≤ 0.05). The results are expressed as adjusted rate ratios and odds ratios and 95 % confidence intervals. Number of prescriptions of antifungal medications to treat oral candidiasis, prevalence of hospitalizations, and initial ICS doses were compared between the two matched cohorts through conditional logistic regression (*p* < 0.05).

## Results

### Patient characteristics and demographics

**Fig. 1:**
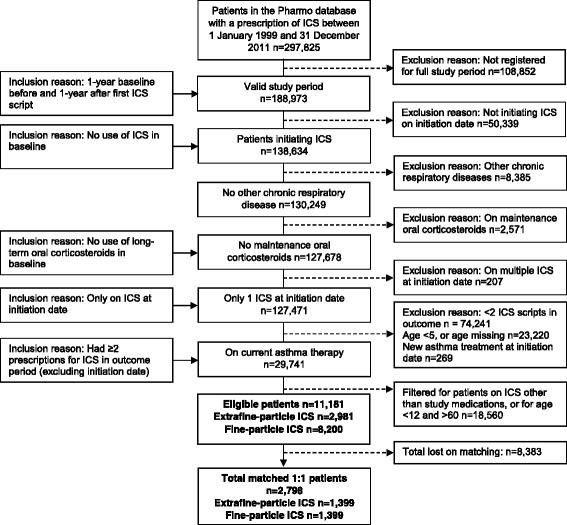
Patient flow chart showing selection of patients in the Pharmo Database Network Patients in the two study cohorts were matched on clinically and demographically significant characteristics. Pharmo: Pharmo Database Network provided by the Pharmo Institute for Drugs Outcome Research (Utrecht, the Netherlands). ICS: inhaled corticosteroid; initiation date: the date when patients received their first prescription of extrafine-particle ICS (ciclesonide or EF-HFA-BDP), or fine-particle ICS (fluticasone or Non-EF-BDP)

Following 1:1 matching, 1399 patients were selected in each of the extrafine-particle ICS and fine-particle ICS cohorts (Fig. 1).

**Fig. 2:**
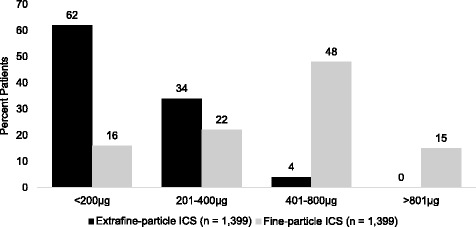
Dose of inhaled corticosteroids prescribed on the initiation date*. Reported doses are the actual dose for extrafine-particle ICS (ciclesonide or EF-HFA-BDP) and the fluticasone-equivalent dose for fine-particle ICS (fluticasone and Non-EF-BDP). * Initiation date: the date when patients received their first prescription of extrafine-particle ICS (ciclesonide or EF-HFA-BDP), or fine-particle ICS (fluticasone or Non-EF-BDP)

Within the extrafine-particle cohort, 712 patients (51 %) were prescribed ciclesonide, 687 (49 %) EF-HFA-BDP. Median (interquartile range [IQR]) age for patients in each of the matched cohorts was 43 years. Median (IQR) ICS doses (μg) were 160 (160–320) for the extrafine-particle cohort versus 500 (250–500) for the fine-particle cohort (*p* < 0.001) (fluticasone-equivalents) (Fig. 2).

**Fig. 3:**
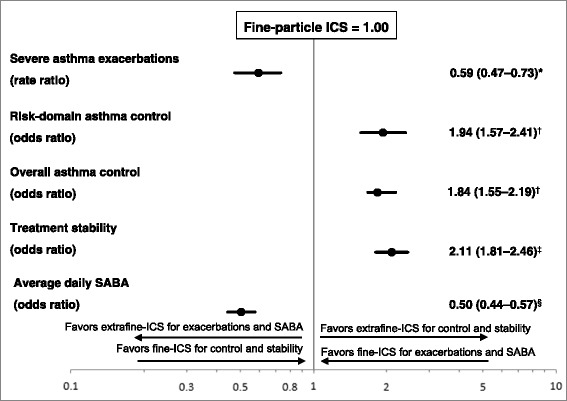
Adjusted rate and odds ratios (95 % CI) for co-primary and secondary outcomes. ICS: inhaled corticosteroid; SABA, short-acting β2-agonist. *Adjusted for baseline exacerbations (ATS/ERS Definition, categorized), evidence of GERD and baseline asthma prescriptions (categorized); ^†^Adjusted for baseline Risk Domain Asthma Control, evidence of GERD and asthma diagnosis; ^‡^Adjusted for evidence of rhinitis, evidence of GERD and baseline Risk Domain Asthma Control; ^§^Adjusted for baseline SABA daily dose

The two study cohorts were similar for the study asthma control measures and prescriptions for SABA (Table 1). However, at baseline, patients in the extrafine-particle cohort received more diagnoses for rhinitis and gastro-oesophageal reflux disease, more prescriptions for acute oral corticosteroids, topical corticosteroid therapy, and had a higher prevalence of asthma-related hospital admissions, than patients in the fine-particle cohort (Table 1). For these specific variables, data were comparable within the extrafine-particle cohort for patients prescribed ciclesonide and EF-HFA-BDP (Table 1).

**Table 1:** Baseline demographic and clinical characteristics for 1:1 matched patients

Baseline Characteristic		Extrafine-particle ICS^a^ (*n* = 1399)	Fine-particle ICS^b^ (*n* = 1399)	*p*-value
Sex, male, n (%)^c^		471 (34)	471 (34)	N/A
Age, median (IQR)^c^		43 (32–53)	43 (32–52)	0.091
Year of initiation date, median (IQR)^c^		2008 (2006–2009)	2008 (2006–2009)	N/A
ICS dose prescribed on initiation date, n (%)				
<200μg/d, n (%)		866 (62)	218 (16)	<0.001
201–400μg/d, n (%)		477 (34)	301 (22)	
401–800μg/d, n (%)		56 (4)	677 (48)	
801μg/d, n (%)		0 (0)	203 (15)	
ICS dose on initiation date, median (IQR)		160 (160–320)	500 (250–500)	<0.001
Average ICS daily dose (μg/d, categorized EF-HFA-BDP/ciclesonide equivalent dose^d^)				
0–200μg/d, n (%)		866 (62)	218 (16)	<0.001
201–400μg/d, n (%)		477 (34)	301 (22)	
401–800μg/d, n (%)		56 (4)	677 (48)	
801μg/d+, n (%)		0 (0)	203 (15)	
Recorded comorbidity, n (%)				
Rhinitis diagnosis, n (%)		623 (45)	536 (38)	<0.001
GERD diagnosis, n (%)		513 (37)	437 (31)	0.001
Paracetamol script, n (%)		28 (2)	47 (3)	0.020
NSAID script, n (%)		219 (16)	215 (15)	0.834
Topical corticosteroid treatment, n (%)		433 (31)	374 (27)	0.014
Oral candidiasis diagnosis, n (%)		14 (1)	11 (1)	0.533
Rhinitis diagnosis, n (%)	Ciclesonide	18 (1)	N/A	N/A
	EF-HFA-BDP	21 (1)		
GERD diagnosis, n (%)	Ciclesonide	0 (0)	N/A	N/A
	EF-HFA-BDP	1 (1)		
Topical corticosteroid treatment, n (%)	Ciclesonide	207 (15)	N/A	N/A
	EF-HFA-BDP	226 (16)		
Respiratory Medications				
Acute oral corticosteroid prescriptions, <1, n (%)		101 (7)	120 (9)	0.001
Acute oral corticosteroid prescriptions, <1, n (%)	Ciclesonide	47 (3)	N/A	N/A
	EF-HFA-BDP	54 (4)		
SABA daily dose, n (%)^c^				
0μg/d, n (%)		953 (68)	953 (68)	N/A
200μg/d, n (%)		57 (4)	57 (4)	
201μg/d, n (%)		40 (3)	40 (3)	
SABA prescriptions, n (%)				
0, n (%)^c^		953 (68)	953 (68)	0.251
1, n (%)		281 (20)	267 (19)	
2+, n (%)		165 (12)	179 (13)	
Prior therapy				
LABA, n (%)^c^		21 (2)	21 (2)	N/A
LTRA, n (%)^c^		1 (0)	1 (0)	N/A
Asthma control				
Risk-domain asthma control, n (%)^c^		1275 (91)	1275 (91)	N/A
Overall asthma control, n (%)^c^		1241 (89)	1241 (89)	N/A
Severe exacerbations, n (%)^c^				
0		1275 (91)	1275 (91)	N/A
1		109 (8)	109 (8)	
2+		15 (1)	15 (1)	
Asthma-related hospital admissions, n (%)		23 (2)	4 (0)	0.001
Asthma-related hospital admissions, n (%)	Ciclesonide	11 (1)	N/A	N/A
	EF-HFA-BDP	12 (1)		

*GERD*, gastro-oesophageal reflux disease; *ICS*, inhaled corticosteroid; *LABA*, long-acting β2-agonist; *LTRA*, leukotriene receptor antagonist; *N/A*: not applicable; *SABA*, short-acting β2-agonist; initiation date: the date when patients received their first prescription of extrafine-particle ICS (ciclesonide or EF-HFA-BDP), or fine-particle ICS (fluticasone or Non-EF-BDP)

^a^Extrafine-particle ICS: ciclesonide; hydrofluoroalkane beclomethasone dipropionate (EF-HFA-BDP)

^b^Fine-particle ICS: non-extrafine-particle beclomethasone dipropionate (Non-EF-BDP); fluticasone propionate

^c^Matching variable

^d^Average ICS daily dose calculated as [(number of inhalers * doses per inhaler / 365) * µg strength]

### Outcomes

Patients who received extrafine-particle ICS had 40 % lower rates of severe exacerbations in the year following the initiation date than those receiving fine-particle ICS therapy, following adjustment for residual confounders (adjusted rate ratio [95 % CI], 0.59 [0.47–0.73]) (Table 2). In addition, patients in the extrafine-particle cohort had greater odds of achieving risk-domain asthma control, overall asthma control, and treatment stability, and had lower odds of being in a higher dose category of SABA (adjusted rate ratio [95 % CI], 0.50 [0.44–0.57]) (Table 2).

**Table 2:** Outcome measures for matched groups 1 year following date of first ICS prescription

Outcome	Extrafine-particle ICS^a^ (*n* = 1399)	Fine-particle ICS^b^ (*n* = 1399)	*p*-value
Asthma control			
Risk-domain asthma control, n (%)	1236 (88)	1129 (81)	<0.001
Overall asthma control, n (%)	1091 (78)	934 (67)	<0.001
Severe exacerbations, n (%)			
0, n (%)	1236 (88)	1129 (81)	<0.001
1, n (%)	108 (8)	184 (13)	
2+, n (%)	55 (4)	86 (6)	
Treatment stability, n (%)	809 (58)	560 (40)	<0.001
Prescribed average daily dose of SABA, μg/d, n (%)			
0 μg/d, n (%)	686 (49)	461 (33)	<0.001
1–100 μg/d, n (%)	326 (23)	343 (25)	
101 μg/d+, n (%)	387 (28)	595 (43)	
1+ prescriptions of antifungal for candidiasis, n (%)	48 (3)	45 (3)	0.753
1+ asthma-related hospital admissions, n (%)	19 (1)	16 (1)	0.613
Respiratory medications in outcome period			
Courses of acute oral corticosteroids, n (%)			
0, n (%)	1246 (89)	1135 (81)	<0.001
1, n (%)	102 (7)	182 (13)	
2+, n (%)	51 (5)	82 (6)	
SABA prescriptions, n (%)			
0, n (%)	686 (49)	461 (33)	<0.001
1, n (%)	245 (18)	235 (17)	
2, n (%)	180 (13)	218 (16)	
3+, n (%)	288 (21)	485 (35)	
ICS prescriptions (including initial prescription)			
2, n (%)	0 (0)	0 (0)	0.022
3, n (%)	462 (33)	520 (37)	
4+, n (%)	937 (67)	879 (63)	
Average ICS daily dose (μg/d, fluticasone-equivalents^c^)			
0–150 μg/d, n (%)	460 (33)	263 (19)	<0.001
151–250 μg/d, n (%)	484 (35)	416 (30)	
251–450 μg/d, n (%)	358 (26)	401 (29)	
451 μg/d+, n (%)	97 (7)	319 (23)	
Average ICS daily dose (μg/d, EF-HFA-BDP/ciclesonide equivalent dose^d^), median (IQR)	185 (132–290)	272 (178–410)	<0.001
Average ICS daily dose (μg/d, EF-HFA-BDP/ciclesonide equivalent dose^d^)			
0–150 μg/d, n (%)	460 (33)	263 (19)	<0.001
151–250 μg/d, n (%)	484 (35)	416 (30)	
251–450 μg/d, n (%)	358 (26)	401 (29)	
451 μg/d+, n (%)	97 (7)	319 (23)	
LABA prescriptions in outcome period	770 (55)	875 (63)	<0.001
LTRA prescriptions in outcome period	83 (6)	47 (3)	0.001

*GERD* gastro-oesophageal reflux disease, *ICS* inhaled corticosteroid, *LABA* long-acting β2-agonist, *LTRA* leukotriene receptor antagonist, *N/A* not applicable, *SABA* short-acting β2-agonist

^a^ Extrafine-particle ICS: ciclesonide (Alvesco®); hydrofluoroalkane beclomethasone dipropionate (EF-HFA-BDP [Qvar®])

^b^ Fine-particle ICS: non-extrafine-particle beclomethasone dipropionate (Non-EF-BDP); fluticasone propionate (FP)

^c^ Fluticasone-equivalent dose for fine-particle ICS (FP and Non-EF-BDP) delivered via a pressurized metered dose inhaler

^d^ EF-HFA-BDP/ciclesonide equivalent dose for extrafine-particle ICS delivered via a pressurized-metered dose inhaler

During the outcome year, patients in the extrafine-particle cohort received fewer prescriptions for other respiratory drugs (oral corticosteroids, SABA and LABA) compared with the fine-particle cohort, although more patients in the extrafine-particle cohort were prescribed LTRAs during the outcome period than the fine-particle cohort (6 % and 3 % in extrafine-particle versus fine-particle ICS cohorts, respectively; p = 0.001) (Table 2). Prescriptions of medications for treating oral candidiasis in the outcome period were not significantly different between the two study cohorts. Although patients in the extrafine-particle cohort had significantly higher asthma-related hospital admissions in baseline (Table 1), in the year following the date of first prescription of ICS therapy, this difference was not evident (Table 2).

## Discussion

In this real-life population-based study, matched patients initiating extrafine-particle ICS had significantly lower rates of severe exacerbations and significantly higher odds of achieving asthma control and treatment stability than those prescribed fine-particle ICS. Notably, the prescribed doses of extrafine-particle ICS were lower than fine-particle ICS at the initiation date (median dose, 160 vs. 500 μg per day, fluticasone-equivalents). In addition, patients prescribed extrafine-particle ICS showed lower odds of being prescribed higher doses of short-acting β2 agonists. Outcome data showed no significant differences between the study cohorts for medications prescribed for treating oral candidiasis, and for asthma-related hospital admissions. These findings suggest a significant improvement in asthma control for patients prescribed extrafine-particle ICS with unstable asthma. Such patients would typically be excluded from traditional RCTs.

Possible mechanisms to account for the superior effectiveness of extrafine-particle ICS observed in this study include improved airway drug deposition and distribution, including distally in the small airways, and improved inhaler device tolerance. These are each discussed in turn below.

Aerosol particle size now appears therapeutically important for controlling asthma symptoms [14]. Evidence indicates that fine-particle ICS with a MMAD of <5 μm, but ≥2 μm, show lower total lung distribution and deposition than extrafine-particles with a MMAD <2 μm, which can deposit more in the small airways [1, 2, 15]. More effective control of small airway inflammation might contribute to improved asthma control [15].

Poor device technique by patients is a common problem observed by clinicians. Asthma control worsens as the number of mistakes in device technique increases [16]. The combination of fine-particle ICS with poor device technique can lead to some of the ICS settling in the oropharynx, resulting in side-effects such as oropharyngeal candidiasis. However, lung deposition of EF-HFA-BDP remains adequate even in patients with poor inhaler device technique [1]. This may be due to pMDI design incorporating hydrofluoroalkane propellant that produces a softer, warmer and longer duration spray. This particular method of delivery has proven more tolerant of poor inhaler technique than some metered dose inhalers emitting fine-particle ICS [1]. Tolerance to inhalation errors may in turn promote improved adherence to therapy [14].

This study has several strengths. To ensure all potentially relevant variables for characterizing patients were included, and that the key outcomes of interest could be evaluated, both the statistical analysis plan, study population and outcomes were conceived prior to any analyses [11]. Imitating traditional RCT design, the study included an initiation date marking therapy initiation. The study inclusion and exclusion criteria were selected to minimize potential confounding factors such as other asthma therapies, and to identify patients from a large clinical population receiving initial ICS therapy. Patient matching was used to adjust for demographic and clinical differences between the study cohorts. An advantage of 1-year baseline and outcome periods is that this time-period allows for natural seasonal changes in respiratory disease, and for recording infrequent clinical events such as exacerbations and asthma-related hospitalizations [11]. The results confirm both the study hypothesis and similar findings observed in real-life asthma control studies with patients in the UK and US [8–10]. This seems to suggest that the present findings can be extrapolated to healthcare systems and prescribing patterns in different countries. Collectively, these studies provide balance to findings reported in some RCTs that extrafine-particle ICS is only equally effective as fine-particle ICS in achieving asthma control [3, 4].

However, as with all real-life comparative database studies, this study has its limitations as well. Limitations could derive from using the Pharmo Database Network, including disease misclassification biases from the almost exclusive use of “asthma prescriptions” to individuate patients with asthma, and the impossibility to adjust for all potential confounders, such as potential confounding by severity for factors indiscernible from patient records or patient-reported outcomes. Asthma diagnosis data, defined as a General Practice recorded diagnosis for asthma (ICPC code R96), were available in the General Practice database for only 12.2 % of the study population. In addition, the results of this study only apply to healthier patients who survive at least 1 year following prescription date. Therefore, this study cannot exclude potential survivor bias [11, 13, 17].

Although specific to respiratory disease, this study could not accurately assess lung function, or symptom control. However, short-acting β2 agonist prescriptions were included in the study as a substitute for asthma symptoms in the ‘overall asthma control’ measure, because short-acting β2 agonist use reflects symptom control [18]. The control cutoff point of a mean short-acting β2 agonist use of ≥2 puffs per day corresponds to the level 2 category (2 of 4, with 1 being the best controlled) of the validated approach of Schatz et al. [18] for short-acting β2 agonist canister dispensing to assess asthma symptom control. During the study period, 1399/2981 (47 %) eligible patients initiating extrafine-particle ICS therapy were matched, and 1399/8200 (17 %) of those initiating fine-particle ICS were matched, possibly indicating that patients in neither cohort were representative of those who initiate extrafine-particle or fine-particle ICS therapy in the Netherlands. Finally, although smoking status was not reported in the study due to poor availability within the Pharmo Database Network, possible COPD patients were included (e.g., patients prescribed long-acting muscarinic agents in addition to ICS), but constituted only 1.8 % of the total study population.

## Conclusions

This study in a real-life population shows that extrafine-particle ICS are associated with better asthma control and better odds of therapy stability than fine-particle ICS, at significantly lower prescribed doses, despite patients having otherwise similar baseline characteristics after exact matching. These results for a Dutch population confirm relevant real-life asthma studies conducted in the UK and US, and seem generalizable to countries with different healthcare systems and prescribing habits. The findings also provide balance to findings reported in some randomized asthma RCTs. The study applied reliable prescribing and medical information recorded in a well-maintained database for a diverse clinical population, a population including those at high risk of exacerbations, with comorbid rhinitis, smokers and non-smokers. Although patients prescribed extrafine-particle ICS showed signs of more severe asthma, they had better asthma control at lower ICS doses than patients prescribed fine-particle ICS. Additional comparative effectiveness studies are required to better understand the differential effects of extrafine-particle versus fine-particle ICS in real-life asthma patients, especially regarding possible subgroup specificities.

## Additional file

Additional file 1: Figure S1. Study design. (DOCX 84 kb)

## Abbreviations

EF-HFA-BDP [Qvar®]: extrafine-particle extrafine-particle hydrofluoroalkane beclomethasone dipropionate
FP: fluticasone propionate
GERD: gastro-oesophageal reflux disease
ICS: inhaled corticosteroid
LABA: long-acting β2-agonist
LTRA: leukotriene receptor antagonist
N/A: not applicable
Non-EF-BDP: non-extrafine-particle beclomethasone dipropionate
Pharmo: Pharmo Database Network pharmo database network provided by the Pharmo Institute for Drug Outcomes Research (Utrecht, the Netherlands)
SABA: short-acting β2-agonist
