# Erratum to: Effectiveness of initiating extrafine-particle versus fine-particle inhaled corticosteroids as asthma therapy in the Netherlands

**DOI:** 10.1186/s12890-016-0270-9

**Published:** 2016-07-23

**Authors:** Thys van der Molen, Dirkje S. Postma, Richard J. Martin, Ron M. C. Herings, Jetty A. Overbeek, Victoria Thomas, Cristiana Miglio, Richard Dekhuijzen, Nicolas Roche, Theresa Guilbert, Elliot Israel, Wim van Aalderen, Elizabeth V. Hillyer, Simon van Rysewyk, David B. Price

**Affiliations:** Department of General Practice, University of Groningen, University Medical Center, Groningen, The Netherlands; University of Groningen, University Medical Center Groningen, Department Pulmonary Medicine and Tuberculosis, Groningen, The Netherlands; National Jewish Health and the University of Colorado, Denver, USA; PHARMO Institute for Drugs Outcome Research, Utrecht, The Netherlands; Research in Real Life, Ltd, Cambridge, UK; Radboud University Medical Centre, Nijmegen, The Netherlands; Groupe Hospitalier Cochin, AP-HP and University Paris Descartes (EA2511), Paris, France; Cincinnati Children’s Hospital and Medical Center, Cincinnati, USA; Brigham and Women’s Hospital and Harvard Medical School, Boston, MA USA; Emma’s Children Hospital, Academic Medical Centre, University of Amsterdam, Amsterdam, The Netherlands; Observational & Pragmatic Research Institute Pte, Ltd, Singapore, Singapore; Academic Primary Care, University of Aberdeen, Polwarth Building, Foresterhill, Aberdeen, AB25 2ZD UK

## Erratum

The original version of this article [1] unfortunately contained errors that have since been corrected.

The word “pharmo” has been fully capitalised to “PHARMO” throughout the article.

The reference to Table 2 in the first and second sentence under the Outcomes heading has been replaced with Fig. 3.

Under the Abbreviations heading ‘extrafine-particle’ was repeated, this has been corrected to ‘EF-HFA-BDP [Qvar®]: extrafine-particle hydrofluoroalkane beclomethasone dipropionate’.

The competing interests of Nicolas Roche and Theresa Guibert have been amended.

Academic affiliations for Dirkje S. Postma (2), Richard J. Martin (3), Ron M.C. Herrings (4), Jetty Overbeek (4), and Nicolas Roche (7) have been corrected.

Figure 3 in the online and pdf version did not match, this been amended.
