# Supplementary material for: Effectiveness of initiating extrafine-particle versus fine-particle inhaled corticosteroids as asthma therapy in the Netherlands
Source: BMC Pulm Med. 2016 May 17;16:80. doi: 10.1186/s12890-016-0234-0 (PMC4869182; doi:10.1186/s12890-016-0234-0)
Supplement: Additional file 1: Figure S1. — Study design. (DOCX 84 kb) [file 12890_2016_234_MOESM1_ESM.docx]

**Online Supplement**

***Study design***

**
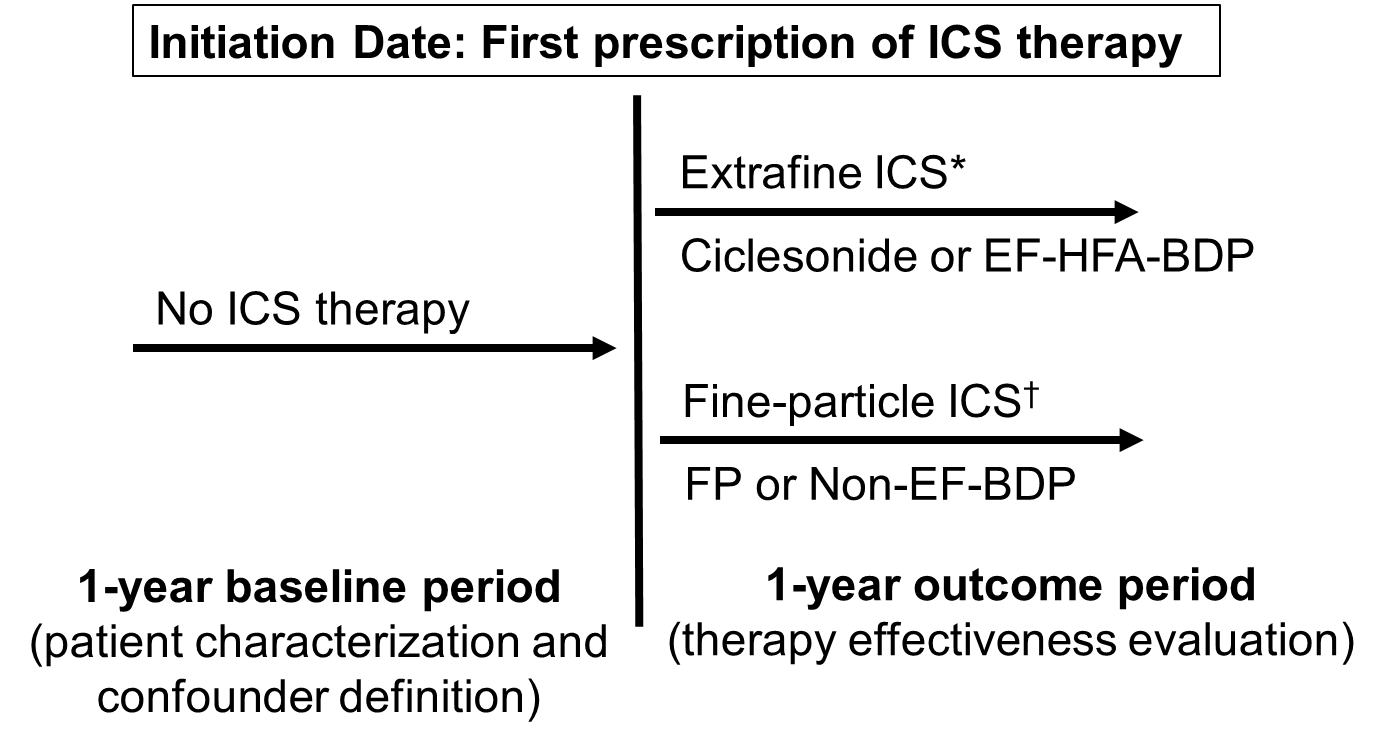
**

**Figure 1. Study design**

EF-HFA-BDP: extrafine hydrofluoroalkane beclomethasone dipropionate; Non-EF-BDP: non-extrafine beclomethasone dipropionate; FP: fluticasone propionate; ICS: inhaled corticosteroid

* Extrafine ICS: mass median aerodynamic diameter (MMAD) = <2μm

† Fine-particle ICS: MMAD = <5 µm but ≥2µm

***Data Source***

The analysis was conducted using datasets from the Pharmo Institute for Drugs Outcome Research (Utrecht, the Netherlands). The Pharmacy database and linked Hospital, Function Laboratory and General Practice databases were used. The study dataset contained patients whose records were linked across the General Practice, Function Laboratories, Pharmacy and Hospital databases. The “ideal” subset within the study population included patients with data on disease diagnosis and medication coding (General Practice), confirmed diagnosis using spirometry (Function Laboratory), medications dispensed (Pharmacy), and use of secondary care (Hospital). Where it was not possible to link patients in the Pharmacy database to their records in the General Practice database, the patient’s medications (Pharmacy data) and secondary care records (Hospital data) were used to infer the patient’s condition.

***Study Outcomes***

***Primary Outcomes***

***Severe exacerbations, risk domain asthma control, overall asthma control***

Emergency department data are not included in the exacerbation definition due to these data not being available in the pharmacy database of PHARMO, but emergency department events likely are recorded by acute oral corticosteroid use. Acute oral corticosteroid use associated with asthma exacerbation treatment is defined as: (1) all courses that are definitely not maintenance therapy, and/or, (2) all courses where dosing instructions suggest exacerbation treatment (e.g. 6,5,4,3,2,1 reducing, or 30μg as directed). Maintenance therapy is defined as no decreasing-dose instructions and daily dose <10μg/day of Prednisolone or prescriptions for 1μg Prednisolone tablets per day, and overall script coverage of more than 25% of days in a year. Where ≥1 oral corticosteroid course or hospitalization occurs within 2 weeks of each other, these events are considered to result from the same exacerbation (and only counted once). Both ‘risk domain asthma control’ and ‘overall asthma control’ definitions do not include the criterion of ‘absence of evidence of GP consultations for LRTI’ due to insufficient linked GP data in the pharmacy database.

***Secondary outcomes***

***SABA usage***

Average daily SABA dosage during outcome year was calculated as average number of puffs per day over the year multiplied by strength (in μg) and categorized as: 0, 1–150, 151–300, >300μg.

***Exploratory outcomes***

***Asthma-related hospital admissions***

The total number of asthma-related hospitalizations per patient and the rate ratio for the average number of hospitalizations per patients in the year following the date of first prescription of ICS (initiation date). *Definite hospitalizations* are hospitalizations coded with a lower respiratory code, including asthma and LRTI codes; or a generic hospitalization read code recorded on the same day as a Lower Respiratory Consultation. Lower Respiratory Consultations consist of lower respiratory read codes (including asthma, COPD and LRTI read codes); asthma/COPD review codes excluding any monitoring letter codes; asthma monitoring. *Definite and probable hospitalizations* include hospitalizations occurring within a 7-day window (either side of the hospitalization date) of a lower respiratory read code.

***Statistical analysis***

Exploratory data analysis was conducted for all baseline and outcome variables.

***Baseline analysis***

***Presentation of results***

The results of the baseline comparisons are presented as p-values. Differences between treatment groups were considered possibly important if p<0.10. Variables meeting this criterion were examined for co-linearity and clinical importance to select those used as potential confounders in the regression modelling of outcomes.

***Predictors of outcomes***

Multivariate analyses were carried out using the full dataset to identify baseline variables that were predictive (p<0.05) of each outcome variable during the outcome period. These were considered as potential confounders when modelling the outcome variables, as follows:

***Predictive variables for severe exacerbations (based on the ATS/ERS Task force definition) rates*:** year of first ICS prescription, gender, beta blockers, number of asthma scripts (categorized), baseline severe exacerbations (categorized) and baseline risk domain asthma control.

***Predictive variables for asthma control*:** age, year of first ICS prescription, gender, asthma diagnosis, evidence of rhinitis, evidence of GERD, evidence of cardiac disease, eczema diagnosis and/or topical steroids, pneumonia diagnosis, pulmonary disease, NSAID use, tricyclics, short-acting anticholinergic (SAAC) use, LAMA use, theophylline use, baseline severe exacerbations (categorized), number of allergy scripts (categorized), number of asthma scripts (categorized), SABA daily dosage (categorized), number of SABA scripts (categorized), baseline risk domain asthma control.

***Predictive variables for overall asthma control:*** age, gender, year of first ICS prescription, asthma diagnosis, evidence of rhinitis, evidence of GERD, evidence of cardiac disease, NSAIDs, beta blockers, tricyclics, eczema diagnosis and/or topical steroids, cancer diagnosis, pulmonary disease, baseline severe exacerbations (categorized), number of allergy scripts (categorized), number of asthma scripts (categorized), number of SABA inhalers (categorized), number of SABA scripts (categorized), SABA daily dosage (categorized), baseline overall asthma control, risk domain asthma control, SAAC use, LAMA use, theophylline use and baseline LABA treatment therapy.

***Predictive variables for treatment stability:*** age, gender, year of first ICS prescription, asthma diagnosis, evidence of rhinitis, evidence of GERD, evidence of cardiac disease, NSAIDs, beta blockers, tricyclics, eczema diagnosis and/or topical steroids, number of allergy scripts (categorized), SABA daily dosage (categorized), baseline risk domain asthma control, baseline severe exacerbations (categorized), Baseline SAAC treatment therapy , Baseline LAMA treatment therapy, number of SABA scripts (categorized) and number of asthma scripts (categorized).

***Predictive variables for SABA usage rates*:** age, gender, year of first ICS prescription, asthma diagnosis, evidence of cardiac disease, beta blockers, statins, tricyclics, eczema diagnosis and/or topical steroids, number of allergy scripts (categorized), SABA daily dosage (categorized), baseline overall asthma control, Baseline SAAC treatment therapy, baseline theophylline treatment therapy, baseline SABA treatment therapy, number of SABA scripts (categorized), number of SABA inhalers and number of asthma scripts (categorized).

***Correlations***

Spearman correlation coefficients were calculated between all potential confounders to determine strengths of linear relationships between variables. The correlation coefficients were considered to identify pairings of variables that might present collinearity issues at the modelling stage. In general, collinearity was considered an issue for relationships with rank correlation coefficients greater than 0.30.

***Outcome analysis***

A comparison of treatment cohorts using the matched datasets was conducted making necessary minimal adjustments for other baseline confounders. Outcome results are provided unadjusted and adjusted for baseline residual confounders for each primary and secondary outcome.

***Primary outcomes analysis***

The total number of *asthma exacerbations* (ERS/ATS definition) in the outcome period were separately compared between cohorts using a conditional Poisson regression model to obtain estimates of relative severe exacerbation rates.

The odds of achieving risk domain asthma control**/**overall asthma control were compared between matched treatment groups using conditional binary logistic regression model.

The models used empirical standard errors (for more conservative confidence interval estimations) and adjustments were made for potential baseline confounders.

***Secondary outcome analysis***

The odds of being prescribed higher dosage of SABA and of addition of new therapy were compared between matched treatment groups using conditional binary logistic regression model.
